# Osteoarthritis and frailty in elderly individuals across six European countries: results from the European Project on OSteoArthritis (EPOSA)

**DOI:** 10.1186/s12891-015-0807-8

**Published:** 2015-11-17

**Authors:** Maria Victoria Castell, Suzan van der Pas, Angel Otero, Paola Siviero, Elaine Dennison, Michael Denkinger, Nancy Pedersen, Mercedes Sanchez-Martinez, Rocio Queipo, Natasja van Schoor, Sabina Zambon, Mark Edwards, Richard Peter, Laura Schaap, Dorly Deeg

**Affiliations:** Preventive Medicine and Public Health, Unit of Primary Care and Family Medicine, Universidad Autonoma de Madrid, Madrid, Spain; IdiPAZ, Instituto de Investigacion de La PAZ, Madrid, Spain; EMGO Institute for Health and Care Research, Department of Epidemiology and Biostatistics, VU University Medical Center, Amsterdam, Netherlands; Medicine and Surgical Sciences. Institute of Neuroscience, University of Padova, Padua, Italy; MRC Epidemiology Resource Centre, University of Southampton, Southampton, UK; Bethesda Geriatric Clinic, University of Ulm, Ulm, Germany; Medical Epidemiology and Biostatistics, Karolinska Institutet, Stockholm, Sweden

**Keywords:** European, Older people, Osteoarthritis, Frailty, Prevalence

## Abstract

**Background:**

Osteoarthritis (OA) is the most common cause of disability in the elderly. Clinical frailty is associated with high mortality, but few studies have explored the relationship between OA and frailty.

The objective of this study was to consider the association between OA and frailty/pre-frailty in an elderly population comprised of six European cohorts participating in the EPOSA project.

**Methods:**

Longitudinal study using baseline data and first follow-up waves, from EPOSA; 2,455 individuals aged 65-85 years were recruited from pre-existing population-based cohorts in Germany, Italy, the Netherlands, Spain, Sweden and the United Kingdom. Data were collected on clinical OA at any site (hand, knee or hip), based on the clinical classification criteria developed by the American College of Rheumatology (ACR). Frailty was defined according to Fried's criteria. The covariates considered were age, gender, educational level, obesity and country. We used multinomial logistic regression to analyse the associations between OA, frailty/pre-frailty and other covariates.

**Results:**

The overall prevalence of clinical OA at any site was 30.4 % (95 % CI:28.6-32.2); frailty was present in 10.2 % (95 % CI:9.0-11.4) and pre-frailty in 51.0 % (95 % CI:49.0-53.0). The odds of frailty was 2.96 (95 % CI:2.11-4.16) and pre-frailty 1.54 (95 % CI:1.24-1.91) as high among OA individuals than those without OA. The association remained when Knee OA, hip OA or hand OA were considered separately, and was stronger in those with increasing number of joints.

**Conclusions:**

Clinical OA is associated with frailty and pre-frailty in older adults in European countries. This association might be considered when designing appropriate intervention strategies for OA management.

## Background

Osteoarthritis (OA) is thought to be the most prevalent chronic joint disease in the world and one of the most common sources of pain and disability in the elderly [1]. Half of the world's population aged 65 and older suffer from OA, and 80 % of people with symptomatic OA have limitations in movement, while 25 % cannot perform their normal daily activities [2]. The prevalence of OA varies widely depending on the whether the criteria adopted are based on self-report, clinical report and/or radiologic imaging [3–8]. It clearly increases with age, and may even triple in frequency in persons age 70 or over and in those with obesity [3, 4].

Because OA occurs in older adults who also have age-related changes in muscle, bone, fat and the nervous system, it is likely that a more general and systemic approach will be needed to better understand the link between aging and OA [9].

Frailty is a physiological state characterized by the deregulation of multiple physiologic systems of an aging organism determining the loss of homeostatic capacity, which exposes the elderly to disability, diseases, and finally death [10–12]. The clinical phenotype of frailty manifests as multi-system pathologies characterized by low physical activity, global weakness with low muscle strength, exhaustion, overall slowness and loss of weight [10, 11]. The worldwide prevalence of frailty ranges between 6.9 % and 42.6 % [10, 13–17]. There is also a documented heterogeneity in the quality of aging among different geographic areas, which suggests the need for a frailty classification approach providing population-specific results [15]. Pre-frailty occurs at an earlier stage of the frailty spectrum and is associated with the later development of frailty. Thus, pre-frailty might be a better target of screening and implementation of early interventions. [12, 17]

Frailty as a geriatric syndrome it has its pathophysiological substrate in sarcopenia [18]; it involves loss of functionality and is a prognostic factor for disability [16]. OA is not purely a mechanical problem. In addition to age, genetic and nutritional factors are also important; obesity predisposes individuals to OA both for mechanical reasons and through inflammatory or metabolic mechanisms [4, 8]. Some studies have found a relationship between OA and frailty, using different diagnostic criteria in both processes [19–21] but, to our knowledge, in Europe there are no population-based studies that relate the two concepts. The EPOSA project is a population-based study using pre-harmonized data across six European countries on older community-dwelling persons aged 65 to 85 years, and it includes clinical data on OA and frailty [22]. This project provides an opportunity for in-depth study of the association between OA and frailty across Europe in an elderly population.

Accordingly, the objective of this study was to consider the association between OA and frailty/pre-frailty in an elderly population comprised of six European cohorts participating in the EPOSA project.

## Methods

### Study design and participants

The EPOSA project involves six cohort studies, each performed in a different country: Germany (The study on Activity and Function in the Elderly in Ulm, ActiFE-Ulm), the United Kingdom (UK) (Hertfordshire Cohort Study, HCS), the Netherlands (Longitudinal Aging Study Amsterdam, LASA), Italy (Godega di Sant’Urbano, Veneto Region), Spain (Ageing in Peñagrande), and Sweden (Swedish Twin Register). Random samples from these population-based cohorts were included. In each cohort, around 750 potential participants were contacted with the aim of recruiting 500 participants. In Italy, a new sample was drawn, with recruitment procedures and age/sex-distributions similar to those in the other studies. A total of 2,942 respondents (response rate ranging from 64.6 % to 82.2 %, averaging 72.8 %) were included in the EPOSA baseline study. The overall age range was 65-85 years (with oversampling of the oldest respondents) in all cohorts except for the UK, in which the age range was 71-79 years.

A detailed description of the study design and data collection of the EPOSA study is described elsewhere [22]. All participants completed an informed consent. For all six countries, the study design and procedures were approved by the Medical Ethics committee of the respective centers (Germany: Ethical Committee of Ulm University; the Netherlands: Medical Ethical Committee of the VU University Medical Center; Spain: Ethic Committee for Clinical Research of University Hospital La Paz of Madrid; Sweden: Ethics Board of Karolinska Institutet; UK: The Hertfordshire Research Ethics Committee; Italy: Comitatio etico ULSS7).

It is a longitudinal study in which all the variables were collected primarily from March to November 2011, except for frailty which was collected one year later (between March and November 2012). The study population for the present analysis was made up of 2,455 individuals who participated in the baseline and the follow-up waves. 487 baseline participants (16.6 %) could not be included because they had died, were untraceable, or declined to participate one year later. The proportion of people aged 80-85 and of women in this group of non-respondents was higher than in participants (28.1 % vs 15.4 %, for age and 57.5 % vs 50.8 % for sex, respectively). The proportion of obese people in both samples was similar. A standardized questionnaire was applied and a clinical examination was performed. Participants were visited in their homes by trained research nurses, except for Italy and Spain, where participants were examined in a health care centre, and only disabled persons were visited in their home.

### Study variables/ measures

Data were collected on the following variables:

#### Clinical osteoarthritis

Algorithms for clinical OA were developed based on the clinical classification criteria developed by the American College of Rheumatology (ACR) [23]. Algorithms were specified both for site-specific OA (knee, hip and hand, respectively) and **Clinical OA at any site** (any of these three joints). The clinical diagnosis of **knee OA** was based on both history and physical examination: pain in the knee was evaluated by the Western Ontario and McMaster Universities OA Index (WOMAC) pain subscale score [24], plus any three of: age 50 or over, morning stiffness lasting <30 min, evaluated by the WOMAC stiffness subscale (score from ‘mild’ to ‘extreme’); crepitus on active motion in at least one side; bony tenderness in at least one side; bony enlargement in at least one side, and no palpable warmth of synovium in both knees. The clinical diagnosis of **hip OA** was based on both history and physical examination: pain in the hip was evaluated by the WOMAC pain subscale score, plus all of: pain associated with hip internal rotation in at least one side; morning stiffness lasting <60 min, evaluated by the WOMAC stiffness subscale (score from ‘mild’ to ‘extreme’); and age 50 or over. The clinical diagnosis of **hand OA** was based on both history and physical examination: pain, aching or stiffness of the hand was evaluated by the Australian/Canadian OA Hand Index (AUSCAN) pain and stiffness subscale [25]; plus any two of: hard tissue enlargement of two or more of the 2nd and 3rd distal interphalangeal (DIPs), 2nd and 3rd proximal interphalangeal (PIPs), 1st carpometacarpal (CMC) joints of at least one hand; hard tissue enlargement of two or more DIPs of at least one hand; deformity of at least one of the 2nd and 3rd DIPs, 2nd and 3rd PIPs, 1st CMC joints of at least one hand. Swelling of the metacarpophalangeal (MCP) joints, which is also included in the ACR classification criteria as a control to exclude rheumatic arthritis, was only measured in the UK and Germany. The categorical variable **Clinical OA-number of sites** describes the number of joints involved, from 0 a 3 (knee, hip and/or hand)

**Frailty** was measured based on the five criteria proposed by Fried [9], with some adaptation as follows:

1) *Unintentional weight loss (Shrinking)* of ≥5 % in the last year; 2) *Low energy (Exhaustion) b*ased on questions from the Centre for Epidemiologic Studies Depression Scale (CES-D); 3) *Weakness*: Grip strength in the dominant hand measured with a dynamometer (*Jamar*^*R*^*)* and adjusted for body mass index (BMI); 4) *Slowness:* Calculated after walking three meters, adjusted for sex and height. Participants were asked to “walk to the other end of the course as quickly as you can, but do not run” and were timed using a stopwatch. In the UK, the instruction was to “walk to the other end of the course at your usual speed, just as if you were walking down the street to go to a shop”. In Germany, 361 of the participants were measured using a GAITRite® walkway system and were standardised using a stopwatch. Individuals included in the worst quintile in each country were considered to be slow. 5) *Low physical activity*: Kilocalories (kcal) expended per week were calculated based on the Longitudinal Ageing Study Amsterdam (LASA) Physical Activity Questionnaire (LAPAQ) [26], using self-reports about the frequency and duration of walking, cycling, gardening and engaging in sports. The cut-off points used in this case were the worst quintile in the EPOSA sample.

Persons who met at least three criteria were considered to be **frail**, those who met one or two criteria were **prefrail**, and those with none were considered **non-frail**. Accordingly, the variable frailty was divided into three categories.

### Potential co-variables

**Demographic covariates** such as **age, gender** and **education level** were collected. Education was measured by asking for the highest level of education completed and was categorized into “elementary school not completed”, “elementary school completed”, “vocational education/general secondary education”, and “college or university education”.

**Health covariates:** BMI was calculated as weight in kilograms divided by height in squared meters. **Obesity** was defined as BMI of 30 kg/m^2^ or higher. Number of chronic conditions was measured through self-reported presence of the following chronic diseases or symptoms that lasted for at least 3 months or diseases for which the respondent had been treated or monitored by a physician: chronic non-specific lung disease, cardiovascular diseases, peripheral artery diseases, stroke, diabetes, cancer, and osteoporosis. **Comorbidity** was evaluated as the number of diseases, or defined as the occurrence of two or more coexisting conditions.

#### Country covariate

Data from the cohorts of Germany, Italy, the Netherlands, Spain, Sweden and the United Kingdom were collected.

### Statistical methods

As age distribution and gender split varied between the cohorts of different countries, a weighting variable was created for each individual within each country. The weights were calculated per sex and per 5-year age groups, using the formula: W = Pexp/Pobs, where Pobs is the observed proportion of persons in a specific age/gender category in the cohort (n_1_/n), and Pexp is the expected proportion of persons in a specific age/gender category in the population (N_1_/N), taking the European Standard Population in 2010 as the reference population [22]. This technique allowed direct comparisons of the variables across countries.

A descriptive analysis was performed. For the continuous variables such as age, the mean and standard deviation were calculated. Categorical variables were expressed as absolute frequencies and their 95 % confidence intervals (95 % CI).

We calculated the frequency of frailty and OA in the study population and made a descriptive analysis of frailty by demographic (age, gender and educational level) and health-related variables (obesity, comorbidity, and OA). Differences among countries were assessed using Kruskal Wallis for age and the chi-squared test for categorical variables.

The association between OA and frailty was summarized with odds ratios (OR) and their 95 % confidence interval (CI) obtained from logistic regression. Multivariate analyses were conducted using the variable frailty with three categories (no frailty as reference variable, pre-frailty and frailty), as the dependent variable (multinomial logistic regression) and OA as the main independent variable, introducing in the model the co-variables that were associated with frailty at *p* < 0.10 in a bivariate analysis.

We conducted five regression analyses, using the previously described measures of OA (clinical OA at any site, knee OA, hip OA, hand OA and clinical OA-number of sites) in each analysis. All analyses were performed using SPSS for Windows version 19.0.

## Results

A total of 2,455 individuals participated in the baseline and the follow-up waves. The mean age of participants in the pooled data across all countries was 74.0 years (SD: 5.0), 50.8 % were women, 42.1 % had no more than elementary school education, 28.6 % were obese, and 27.5 % presented comorbidity. Clear differences were observed between countries with regard to educational attainment. Over 70 % of participants in the cohorts of Italy and Spain had at most a primary school education, whereas this percentage was 20.9 % in the UK, 21.9 % in Sweden and 24.4 % in the Netherlands. In Germany, half of the participants had reached or exceeded the level of secondary school education (Table 1).

**Table 1:** Characteristics of participants by country

	Overall	Country					
	*N* = 2455	GER	NL	IT	SP	SW	UK
		*N* = 336	*N* = 483	*N* = 319	*N* = 457	*N* = 450	*N* = 410
Age (years)^a^ Mean (SD)	74.0 ± 5.0	74.1 ± 4.9	74.9 ± 5.6	72.8 ± 5.0	74.6 ± 5.4	71.9 ± 4.9	75.2 ± 2.6
	% [95% CI]	% [95% CI]	% [95% CI]	% [95% CI]	% [95% CI]	% [95% CI]	% [95% CI]
Gender female^a^	50.8(48.8-52.8)	39.6(34.4-44.8)	54.9(50.5-59.3)	50.8(45.3-56.3)	47.5(42.9-52.1)	58.7(54.2-63.2)	50.2(45.4-55.0)
Education (≤ elementary)	42.1(40.1-44.1)	48.4(43.1-53.7)	24.4(20.6-28.2)	74.9(70.1-79.7)	71.1(66.9-75.3)	21.9(18.1-25.7)	20.9(17.0-24.8)
Obesity (BMI ≥ 30)	28.6(26.8-30.4)	45.3(40.0-50.6)	26.4(22.5-30.3)	25.4(20.6-30.2)	34.4(30.0-38.8)	16.1(12.7-19.5)	29.0(24.6-33.4)
Comorbidity (≥2 diseases)	27.5(25.7-29.3)	27.4(22.6-32.2)	23.8(20.0-27.6)	33.7(28.5-38.9)	33.2(28.9-37.5)	20.8(17.0-24.6)	28.9(24.5-33.3)

Weighted data to the European standard population in 2010 except^a^

*P*-value <0.001 in all variables between countries

**Fig. 1:**
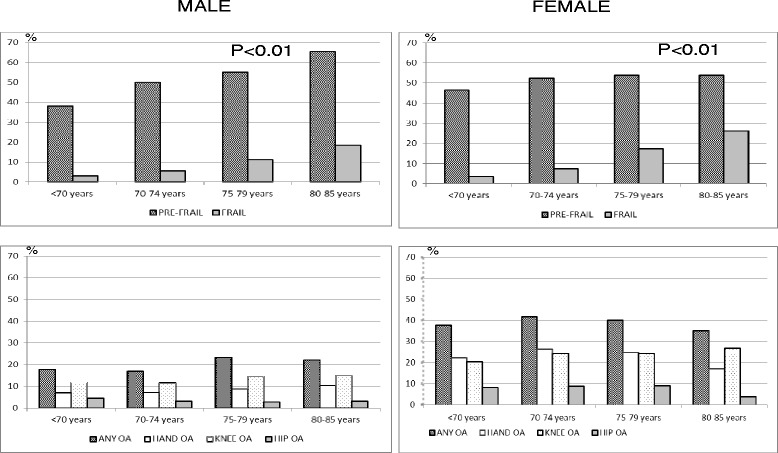
Prevalence of OA and frailty by age and sex in the overall EPOSA sample (*n =* 2455)

The overall prevalence of clinical OA at any site was 30.4 %; 16.3 % had OA of the hand, 5.9 % of the hip, and 19 % of the knee (Table 2). The highest levels of clinical OA at any site were found in Italy (42.3 %), which also had the highest figures for OA of the hand, knee and hip. Germany had the lowest levels, with 19.7 % of clinical OA at any site. Women had a higher frequency of OA than men in all age groups, both when analysed overall and by each OA site (Fig. 1).

**Table 2:** Frequency of OA and frailty status by country (weighted data^a^)

	Overall	GER	NL	IT	SP	SW	UK %	p**
	% [95 % CI]	% [95 % CI]	% [95 % CI]	% [95 % CI]	% [95 % CI]	% [95 % CI]	[95 % CI]	
Clinical OA at any site	30.4(28.6-32.2)	19.7(15.4-24.0)	26.0(22.1-29.9)	42.3(36.9-47.7)	34.7(30.3-39.1)	32.7(28.4-37.0)	26.7(22.4-31.0)	<0.001
Hand OA	16.3(14.8-17.8)	11.8(8.4-15.2)	11.6(8.7-14.5)	21.2(16.7-25.7)	19.5(15.9-23.1)	19.2(15.6-22.8)	14.5(11.1-17.9)	<0.001
Hip OA	5.9(5.0-6.8)	0.7(0.3-1.6)	6.9(4.6-9.2)	13.8(10.0-17.6)	4.7(2.8-6.6)	4.6(2.7-6.5)	5.0(2.9-7.1)	<0.001
Knee OA	19.0(17.4-20.6)	10.4(7.1-13.7)	18.3(14.9-21.7)	25.4(20.6-30.2)	23.5(19.6-27.4)	19.7(16.0-23.4)	15.1(11.6-18.6)	<0.001
Frail	10.2(9.0-11.4)	5.6(3.1-8.1)	10.9(8.1-13.7)	12.2(8.6-15.8)	12.6(9.6-15.6)	5.6(3.5-7.7)	15.4(11.9-18.9)	<0.001
Pre-frail	51.0(49.0-53.0)	48.4(43.1-53.7)	47.1(42.6-51.6)	61.9(56.6-67.2)	56.1(51.5-60.7)	42.2(37.6-46.8)	54.8(50.0-59.6)	<0.001
Non-frail	38.8(36.9-40.7)	46.1(40.8-51.4)	42.1(37.7-46.5)	26.0(21.2-30.8)	31.4(27.1-35.7)	52.2(47.6-56.8)	29.8(25.4-34.2)	<0.001

^a^The weighted data were derived from the European standard population in 2010

**P-value <0.001 in all variables between countries

Frailty was present in 10.2 % of the population, ranging across countries from a prevalence of 5.6 % in Germany and Sweden to 15.4 % in the UK (*p* < 0.001). The overall prevalence of pre-frailty was 51.0 % (Table 2). Both frailty and pre-frailty were higher in women and increased with age in both sexes, with frailty reaching 26.1 % in women aged 80 and over (Fig. 1).

The bivariate analysis showed that all covariables were associated with frailty or pre-frailty at p <0.05.

Table 3 shows the association between OA and pre-frailty and frailty. The crude OR of OA in prefrail and frail people was 1.74 and 3.69, respectively. After adjustment for all the study variables, the OR for OA was reduced to 1.54 for pre-frailty and to 2.96 for frailty. It can be seen that comorbidity and obesity as co-variables in this fully adjusted model are independently associated with both pre-frailty and frailty.

**Table 3:** Association between Osteoarthritis (OA) and pre-frailty/frailty. Multinomial regression

	PRE-FRAILTY	FRAILTY
Osteoarthritis (OA) (crude)	1.74[1.42-2.12]	3.69[2.74-4.97]
OA adjusted for age and sex	1.78[1.45-2.19]	3.91[2.83-5.40]
*OA adjusted for age, sex, education & country*	1.68[1.36-2.08]	3.65[2.63-5.08]
*OA adjusted for age, sex, education, country & comorbidity*	1.60[1.30-1.99]	3.26[2.34-4.56]
*OA adjusted for age, sex, education, country, comorbidity & obesity*	1.54[1.24-1.91]	2.96[2.11-4.16]

Values expressed as Odds Ratios. OR (95 % CIs)

Reference category: non-osteoarthritis

Table 4 shows the fully adjusted OR of the different OA variables used. After adjusting for the confounding variables, including country, the presence of OA was associated with pre-frailty (OR:1.54; 95 % CI:1.24-1.91) and, more strongly, with frailty (OR:2.96; 95 % CI:2.11-4.16). This association was maintained for each of the joints analysed, and the odds of frailty were four times higher when the hip was the affected joint (OR:4.41; 95 % CI:1.41-13.82). The strength of the association increased with the number of affected joints; when OA was present at the same time in all three joints analysed, the odds of pre-frailty were three times higher (OR 2.26; 95 % CI:1.28-8.32) and the odds of frailty were over eight times higher (OR: 8.95; 95 % CI:2.83-28.39).

**Table 4:** Fully adjusted association between Osteoarthritis (OA) in different sites and pre-frailty/frailty status (five multinomial regressions)^a^

	PRE-FRAILTY	FRAILTY
CLINICAL OA AT ANY SITE^b^	1.54 [1.24-1.91]	2.96 [2.11-4.16]
KNEE OA^c^	1.43 [1.04-1.98]	2.08 [1.25-3.46]
HIP OA^c^	1.95 [0.86-4.42]	4.41 [1.41-13.82]
HAND OA^c^	1.50 [1.06-2.12]	2.57 [1.46-4.55]
CLINICAL OA-NUMBER OF SITES		
OA 1 SITE^b^	1.45 [1.14-1.85]	2.47 [1.68-3.63]
OA 2 SITES^b^	1.73 [1.16-2.57]	4.18 [2.42-7.22]
OA 3 SITES^b^	2.26 [1.28-8.32]	8.95 [2.83-28.39]

Values expressed as Odds Ratios. OR (95 % CIs)

^a^OR Adjusted for age, gender, education, country, comorbidity and BMI

^b^Reference category: non-OA at any site

^c^Reference category: non-OA at that site

## Discussion

This study suggests a strong association between OA and frailty that remains after adjusting for socio-demographic and health-related variables and that is maintained when analysing different sites separately (knee, hip, hand). The odds of pre-frailty and frailty were 1.54 and 2.96 times higher among OA than non-OA patients.

Recent publications have found an independent association between hip OA and frailty or pre-frailty in men aged 65 and over [27] and knee OA has been shown to be associated with a greater prevalence and risk of developing frailty [20]. Other studies have found a relationship between frailty and OA measured with subjective criteria [15, 21]. These results suggest common pathophysiological mechanisms underlying both conditions. Certain inflammatory cytokines (IL1, IL-6 and TNFalpha) that are involved in the frailty cycle [18, 28, 29] are increased in OA cartilage as opposed to normal cartilage. The response to growth factors such as IGF1 declines markedly, thus inhibiting maintenance of normal cartilage and promoting the development of OA [18, 28].

The high prevalence of OA among the elderly is well known [3–7]. In our study, 30.4 % of cases had OA in one or more of the joints studied. The prevalence of OA at any site was very high in Italy (42.3 %) and was low in Germany (19.7 %), and these differences were maintained regardless of the joint affected. Although there are demographic differences across countries such as age, sex, educational level, the variability in prevalence rates may be influenced by other factors as climate, health care, lifestyle or environmental factors [22, 30]. The findings of other studies in the developed world are consistent with ours. In the Rotterdam Study the prevalence of OA based on clinical criteria was 16.2 % in men and 20.4 % in women aged 75-84 years [31]. The Johnston County study reported a frequency of 47 % in men and 49.2 % in women aged 70-74 years [32].

The prevalence of frailty is high in the elderly and will increase in the future due to the progressive ageing of the population. Our results show that the overall prevalence of frailty is 10.2 % and of pre-frailty is 51.0 %, which is consistent with other population-based studies of similar characteristics conducted in Europe [14, 21]. The Survey of Health, Ageing and Retirement in Europe (SHARE) [20], which compared frailty across 10 European countries, found a much higher prevalence in the Mediterranean countries (Italy and Spain) and a lower prevalence in Nordic countries like Sweden. Because of the high frequency and the earlier stage of the frailty spectrum, the prefrail constitute a target population requiring action from the health and social services [33, 34].

Obesity is strongly associated with OA and frailty in older people, and in the presence of the three conditions there is a higher risk of functional limitation [19, 27, 35–37]. In our study, obese individuals had a higher risk of being prefrail and frail, as we can see in Table 3. It is important to realize that obesity acts not only as a local biomechanical factor, but as a systemic component [4] and its influence increases with age: Body composition changes with age, even if body mass index (BMI) does not vary, with an increased proportion of fat mass and decrease in lean mass. It is now appreciated that these age-related changes occurring in tissues besides articular cartilage may contribute to the development of OA [1]. However, these changes are much more intense in the presence of obesity, frailty and/or OA and, as noted by several authors, may be due to the development of insulin resistance and the maintenance of chronic inflammatory processes over time [8, 10, 14]. Sarcopenic obesity, a condition in which lean body mass is lost while fat mass may be preserved or even increased [17] has a stronger association with knee OA than non-sarcopenic obesity, indicating the importance of the systemic metabolic effect of obesity in OA [29].

This study has some limitations. Although all participants were recruited from pre-existing community-based cohorts of older individuals, the cohorts may not be representative of their respective countries to the same extend, due to differential attrition. However, these differences may allow hypothesis generation regarding the association between OA and frailty. The non-participation in the 1-year follow-up of 487 baseline individuals may influence the results, but given the higher proportion of women and older individuals, which are variables associated with a higher prevalence of frailty and OA, the results presented would likely have been stronger had we been able to include the non-participants. Another limitation is that the involvement of multiple research centres meant that data collection methods might vary by study site. However, all questionnaires and protocols for examination were undertaken by one team to minimize such problems and OA was diagnosed according to clinical criteria and following a similar methodology in all the participating cohorts. Finally, although the study design is longitudinal, given that the main variables (OA and frailty) are chronic processes, and that the period between baseline and the follow-up wave was very short, we cannot assume the temporal direction of causality in the association detected. Other longitudinal studies over a longer time period are needed to assess the relationship between the incidence of frailty or pre-frailty and the presence of OA.

Due to the high prevalence of OA and frailty in persons aged 65 and over, the strong association between the two processes and the fact that frailty has been considered as a predictor of mid-term mortality in individuals with OA [35], several authors have recommended preventive and therapeutic interventions at the community level. These include, firstly, the early detection of frailty as an important measure of overall health status in the elderly [27]. The frailty score proposed by Fried et al. has biological validity and is easy and inexpensive to measure [14]. Furthermore, OA seems to be associated with worsening of and/or lower recovery from frailty [33]. Thus, we should encourage the use of this scale in the screening of older persons, especially those age 75 and over [38].

Another intervention may be to promote or prescribe appropriate exercise. The practice of regular physical activity that includes both aerobic and resistance exercises tailored to each individual's needs is probably the most efficient intervention to prevent frailty and to delay disability and the adverse events associated with obesity and frailty [34, 39, 40]. Health benefits can be obtained at any point in the evolution of OA and frailty [40].

Finally, weight loss is a priority in the long-term management of obese individuals with OA. Not only does this help lessen joint overload, it also results in decreased fat mass and a proportional increase in lean mass which leads to functional improvement [29]. The Mediterranean diet, rich in olive oil, fruits and vegetables, and with a predominance of polyunsaturated fatty acids, also contributes to improved functionality and quality of life in individuals with osteoarticular pathology [41].

## Conclusion

The prevalence of both OA and frailty/pre-frailty in European countries is high, involving nearly one-third and two-thirds of the elderly, respectively. Clinical OA is strongly associated with frailty and pre-frailty in older adults. Although the association exists with OA at any site, it is stronger with increasing number of joints involved and when the affected joint is the hip. Thus, the key is to implement preventive and therapeutic measures in older persons with OA, especially early detection of frailty and the promotion of physical exercise in all individuals and of fat weight loss in obese individuals.

### Significance and innovations

- OA and frailty are highly prevalent in the elderly population in Europe, with an estimated frequency that ranges between 19.7 % in Germany and 42.3 % in Italy for OA and between 5.6 % in Germany and Sweden and 15.4 % in UK for frailty.
- The odds of frailty is 2.96 (95 % CI:2.11-4.16) and pre-frailty 1.54 (95 % CI:1.24-1.91) as high among OA individuals than those without OA. The association remains when OA of the knee, hip and hand joints are considered separately, and is stronger in those with increasing number of joints involved.
- This association might be considered when designing appropriate intervention strategies for OA management.
